# Ponticelli regimen in idiopathic nephrotic syndrome

**DOI:** 10.4103/0971-4065.53321

**Published:** 2009-04

**Authors:** U. Das, K. V. Dakshinamurty, N. Prasad

**Affiliations:** Department of Nephrology, Nizam's Institute of Medical Sciences, Hyderabad, India

**Keywords:** Idiopathic nephrotic syndrome, Ponticelli regimen, prednisolone, chlorambucil

## Abstract

Various studies have demonstrated that treatment with methyl prednisolone and chlorambucil could increase the chance of remission of idiopathic nephrotic syndrome (INS) of varied histology in patients who do not respond to the conventional treatment. This study was done to assess the safety and efficacy of methyl prednisolone and chlorambucil regimen in patients with various types of glomerulonephritides which were resistant to the usual conventional immunosuppressive drugs. Thirty nine patients were treated between June 1998 and December 2003 with Ponticelli regimen for six months. Twenty three patients (58.98%) were men and 16 (41.02%) were women. Mean age at the onset of NS was 23.59 ± 1.28 (range 10-51) years. Four patients (10.2%) had minimal change disease (MCD), six patients (15.4%) had membranoproliferative glomerulonephritis (MPGN), two (5.1%) had IgA nephropathy, and 18 patients (46.1%) had focal segmental glomerulosclerosis (FSGS). Eleven patients were excluded from the final analysis. Of the remaining 28 patients, mean baseline proteinuria was 3.31 ± 3.09 g/day. Mean baseline plasma albumin was 2.84 ± 1.002 g/dl and mean baseline serum creatinine was 0.87 ± 0.42 mg/dl. At the end of six months of treatment, mean proteinuria was 1.02 ± 0.85 g/day. Mean plasma albumin was 3.69 ± 0.78 g/day, and mean serum creatinine was 0.85 ± 0.26 mg/dl. Mean followup was 13.21 ± 7.7 times in 18.92 ± 12.58 months. At the end of six months of treatment, seven patients (25%) achieved complete remission (CR), 10 patients (35.71%) partial remission (PR), and 11 patients (39.3%) did not show any response to the therapy. Most of the patients in responder group had FSGS (64.70%), whereas in nonresponder group patients had MPGN and mesangioproliferative glomerulonephritis (MesPGN). Out of 13 FSGS cases five (38.46%) achieved CR, six (46.15%) PR, and only two (15.38%) failed to respond. The incidence of side effects was 39.3%. Responders had more side effects than nonresponders (47 vs 27.3%). Methyl prednisolone and chlorambucil therapy (Ponticelli regimen) is safe and efficacious in achieving remission in significant number of INS patients other than membranous nephropathy, without any serious side effect on short term followup. However, a longer followup is required to demonstrate the sustained efficacy and long-term side effect of this regimen.

## Introduction

There is no standard therapy for patients with frequent relapsing or steroid-dependent nephrotic syndrome (NS) with primary glomerulonephritides (GN).[1] Similarly, the optimal approach to steroid resistant idiopathic nephrotic syndrome (INS) is uncertain. Prolonged or repeated steroid therapy can lead to a variety of serious side effects.[2] Achieving remission is an important goal that predicts an excellent long-term prognosis. For over 30 years cyclophosphomide (CYC) and chlorambucil (Chl) have been used to treat children with relapsing steroid sensitive NS.[3] Seventy five to eighty percent of patients with focal segmental glomerulosclerosis (FSGS) are steroid resistant and majority of them slowly progress to endstage renal disease (ESRD)[4] Ponticelli *et al.*, in a long follow up of a randomized controlled study had concluded that six months course of methyl prednisolone (MP) and Chl can increase remission of protienuria and protect from deterioration of renal function in membranous nephropathy. They also observed similar result with the same treatment protocol in steroid resistant NS with FSGS and frequently relapsing (FR) GN with minimal changes disease (MCD) in various studies.[5] Several studies have demonstrated that treatment with MP and Chl could increase the chance of remission in varied histology in patients who do not respond to the conventional treatment.[1]

We conducted this prospective study to evaluate the safety and efficacy of MP and Chl regimen in patients with various types of GN, who were resistant to the usual immunosuppressive drugs.

## Materials and Methods

This was a prospective uncontrolled study. The study group comprised of 39 consecutive patients who were frequent relapsers or steroidresistant NS of varied GN. Informed consent was taken from all patients.

Adequate biopsy specimen for light microscopy and immunofluorescence was obtained from all patients and examined by the pathologist of the institute.

All patients were followed up monthly for one year and bimonthly for next one year. On each visit the following investigations were carried out: Hemoglobin, total leukocyte count, serum total protein and albumin, creatinine, cholesterol and triglyceride, 24 h urinary protein, and also liver function tests once patients were on levamisole. In addition, patients were clinically evaluated for disease activity and complications. Patients were monitored for following side effects: infections, leukopenia, gastric hemorrhage, diabetes, neoplasia, alopecia, anemia, Cushingoid appearance, gastric discomfort, etc.

Statistical analysis was done using Microsoft Excel software. Chi square test and Student's ‘*t*’ test were used. *P* value less than 0.05 was considered as significant.

## Results

The present study was commenced in June 1998. It was a prospective uncontrolled study to assess the safety and efficacy of MP and Chl regimen in patients with various types of GN, who were resistant to the usual conventional immunosuppressive drugs. A total of 39 patients were prospectively recruited. Twenty three patients were men (58.98%) and 16 (41.02%) were women. Of these 33 (84.61%) were SR, five (12.82%) were FR, and one (2.56%) patient was SD. Mean age at the onset of NS was 23.59 ± 11.28 years (range 10-51 years). Mean duration of disease was 31.84 months (1–120 months). The demographic, clinical, and biochemical details, and histopathology of patients at enrollment are shown in Table 1. A total of 11 patients were excluded from the final analysis. Of these, five had FSGS, one had MCD, one had membranoproliferative GN (MPGN), three had mesangioproliferative GN (MesPGN), and one had IgA nephropathy (IgAN). Out of which seven patients were irregular on follow-up and four patients did not tolerate the treatment (three had pneumonia in the second cycle and one had persistent leukopenia and anemia) and were shifted to mycofenolate mofetil (MMF). The age of the patient with persistent leukopenia and anemia was 40 years. A total of 28 patients were included for the final analysis. Mean follow-up of these patients was 13.21 ± 7.7 times in 18.92 ± 12.58 months. At the end of 6 and 12 months, there was a significant reduction of proteinuria (*P* < 0.001) and improvement of plasma albumin from baseline value (*P* < 0.001) [Table 2]. Serum creatinine level before and after therapy was similar. Seven (25%) patients achieved CR and 10 (35.71%) patients achieved PR. Eleven (39.28%) patients did not respond to the treatment. The demographic data, clinical presentation, and baseline biochemical parameters were similar in both responders and nonresponders [Tables 3 and 4]. There was a significant difference between FSGS and other three histopathological groups (*P* < 0.05) by Chi-square test.

**Table 1:** Demographic, clinical, and biochemical details, and histopathology of patients at enrollment

Total no. of patients	39
Sex M/F	23:16
Mean age in years (range)	23.59 ± 11.28 (10-51yrs)
Duration of disease in months (range)	31.84 ± 30.11 (1–120)
S. creatinine	0.87 ± 0.42
Protienuria (mean, g/day)	3.31 ± 3.09
S. albumin (mean, g/dl)	2.84 ± 1
Mean of follow-up in month	13.21 ± 7.7
HTN (%)	24 (61.53)
Hematuria (%)	15 (38.46)
Renal insufficiency (%)	11 (28.2)

**Histopathology**	**No. of patients (%)**

FSGS	18 (46.15)
MCD	4 (10.25)
MPGN	6 (15.38)
MesPGN	9 (23.07)
IgA nephropathy	2 (5.12)

Values are shown as mean ± SE; FSGS: Focal segmental glomerulosclerosis; MCD: Minimal change disease; MPGN: Membranoproliferative glomerulonephritis; MesPGN: Mesangioproliferative glomerulonephritis; HTN: Hypertension

**Table 2:** Response to treatment at the end of six months and on follow up at 12 months and24 months

	Baseline	At 6 months	*P* value	At 12 months	*P* value	24 months
Proteinuria (g/day)	3.31 ± 3.09	1.24 ± 1	0.002	0.81 ± 0.56	0.0003	0.82 ± 1.28
Plasma albumin (g/dl)	2.84 ± 1.002	3.45 ± 0.92	0.02	3.94 ± 0.55	0.04	3.94 ± 0.43
Mean S. creatinine (mg/dl)	0.87 ± 0.42	0.83 ± 0.34	0.622	0.77 ± 0.34	0.157	0.82 ± 0.94
Response						
CR (%)		7 (25)		8 (28.57)		8 (28.57)
PR (%)		10 (35.71)		4 (14.28)		3 (10.71)
NR		11		11		11
Relapse		None		2		1
Loss of f/u		None		3		6

Differences in between responders and nonresponders

**Table 3:** Demographic data and clinical presentation in responders and nonresponders

Variable	Responders	Nonresponders	*P* value
No. of patients	17	11	NS
M:F	11:6	5:5	NS
Mean age (yrs)	22.82 ± 11.18	23 ± 8.8	0.96
Duration of disease (months)	41.64 ± 36.68	23.72 ± 13.29	0.08
Hypertension (%)	8 (47.05)	6 (54.54)	NS
Hematuria (%)	3 (17.64)	5 (45.45)	NS
Renal insufficiency (%)	2 (11.76)	2 (18.18)	NS

NS:Nonsignificant

**Table 4:** Baseline biochemical parameters in responders and nonresponders

Variable	Responders	Nonreponders	*P* value
Proteinuria (g/day)	3.45 ± 2.28	3.28 ± 4.28	0.97
Plasma alb (g/dl)	2.49 ± 0.85	3.48 ± 0.997	0.024
S. creatinine (mg/dl)	1.12 ± 0.58	0.87 ± 0.59	0.97

FSGS responded better than other three groups in our study [Table 5]. Out of 13 FSGS cases, five (38.46%) achieved CR, six (46.15%) had PR, and only two (15.38%) failed to respond, and three patients had nephrotic relapse [Table 6]. The incidence of side effects was 39.28%. Four patients (14.28%) developed respiratory tract infection (RTI). Leukopenia was seen in three patients (10.71%) and was transient, only one patient required treatment with G-CSF. Two (7.14%) patients developed herpes zoster, which was managed with oral acyclovir. Out of total six children, only one had growth retardation. This particular patient had suffered from NS since the age of six years and received steroid therapy since that age every now and then. Drug-induced diabetes mellitus (DM) was observed in one patient [Table 7]. However, three patients discontinued therapy due to severe infection (pneumonia) at the beginning of the treatment and one patient due to persistent leukopenia and anemia. These patients were excluded from the study.

**Table 5:** Histopathological differences in responders and nonresponders

Histopathology	Responders	Nonresponders	Relapse
FSGS (13)	11	2	3
MCD (3)	2	1	none
MPGN (5)	1	4	none
MesPGN (6)	2	4	none
IgA nephropathy (1)	1	Nil	none

**Table 6:** Response of the patients with focal segmental glomerulosclerosis

Response	No. of patients*	Percentage
Complete response	5	38.46
Partial response	6	46.15
Nonresponse	2	15.38

* *n* = 13

**Table 7:** Side effects

Side effects	No. of patients*	Percentage
Respiratory tract infection	4	14.28
Leukopenia	3	10.71
Herpes zoster	2	7.14
Growth retardation	1	3.57
Drug induced diabetes mellitus	1	3.57

* *n* = 11 (39.28%)

As shown in Table 8, out of 17 responders, eight (47%) patients suffered from side effects. In nonresponder group, 3 out of 11 patients (27.27%) suffered from side effects. Side effects were more in responder group [Table 8].

**Table 8:** Differences of side effects between responders and nonresponders

Side effect	Responders	Nonresponders
No. of patients	17	11
RTI (%)	2 (11.76)	2 (18.18)
Leukopenia (%)	3 (17.64)	None
Herpes zoster (%)	2 (11.76)	None
Growth retardation (%)	1 (5.88)	None
Drug-induced DM (%)	None	1 (9.09)

## Discussion

In this study, intravenous MP, Chl, and low-dose prednisolone combination (Ponticelli regimen) was found to be an effective for NS patients resistant to usual immunosuppressive therapy with various types of GN other than membranous nephropathy. There were no significant differences in clinical presentation and demographic data between responders and nonresponders at baseline. Histopathological type was found to have an impact on the outcome. SRNS with FSGS had a favorable outcome as 11 (64.7%) patients had achieved remission at the end of six-month therapy. Published literature regarding the use of this treatment in various types of GN, other than membranous nephrotic syndrome (MNS), was very little. Ponticelli *et al*., showed that, out of total 15 cases with FSGS, CR was observed in 30% patients, PR in 11.1%, and 58.9% patients did not respond.[5] Banfie *et al.*, used Ponticelli protocol in 19 NS patients with FSGS, of which 10 obtained CR in six months.[11] Our result is comparable with these studies.

Mendoza *et al.*, treated 23 NS children with FSGS with i.v. MP pulses plus oral prednisone and cytotoxic drug, but not as per Ponticelli protocol. After a mean follow-up of 55 months 18 patients were without NS and only one patient had progressed to renal failure. In spite of very aggressive approach side effects were relatively mild in that particular series.[12] Similarly Tune *et al.*, used intravenous MP and Chl in an aggressive manner in 32 SR FSGS children, of which 21 had CR on five-year follow-up.[13] Niaudet *et al.*, found that Chl at a cumulative dose of 8 mg/kg is effective in inducing sustained remission in INS.[14] Most of the above mentioned studies found that intravenous MP pulses with alkylating agent and low-dose prednisolone were effective in bringing remission in SR NS with FSGS.

Treatment of MPGN remains elusive. Symptomatic therapy and good control of hypertension (HTN) are important. In nephrotic patients a course of steroid may be tried. In the presence of an extracapillary GN or a superimposed interstitial nephritis, an aggressive treatment with i.v. MP pulses, oral steroid, and CYC may obtain a substantial recovery of renal function in several patients.[1] We tried Ponticelli protocol in five NS patients with MPGN and six patients with MesPGN who were SR. Only 5.88% and 11.76% achieved remission respectively, whereas 23.52% in both classes did not respond to the therapy. So, in our observation, this regimen is partially effective in MPGN and MesPGN. Only a larger sample size will give a better idea.

Out of total three cases of MCD, two patients achieved remission and one did not respond. No article was found in literature regarding use of this treatment in MCD. However in one study Ponticellli *et al.*, had concluded that elderly SR patients with MCD might benefit from a short course of (8–12 weeks) alkylating agent (CYC 2 mg/kg/day or Chl 0.15 mg/kg/day) associated with oral prednisolone at low doses (20–25 mg/day).[1] Schena *et al.*, also found benefit from eight weeks therapy with CYC 2 mg/kg/day or Chl 0.15 mg/kg/day in MCD.[15]

Nephrotic syndrome is uncommon in IgA nephropathy and usually associated with a poor prognosis. Schena *et al.*, in a meta-analysis, showed that the patient with heavy proteinuria benefited from the administration of steroid and/or cytotoxic drug, as 67% of treated patients had CR or PR versus 34% of untreated patients.[16] A special group of patients showed rapid impairment of renal function, for them aggressive treatment with i.v. MP, oral prednisolone, and CYC may be useful.[1] We used this protocol to treat one NS patient with IgA nephropathy with FR who presented with renal impairment. This patient had achieved CR at end of six-month therapy with improvement in renal function.

In the study group, only 11 patients (39.28%) had infection in the form of RTI in four cases (14.28%) and herpes zoster in two cases (10.71%), and all of them recovered with treatment. However, at the initiation of the therapy, four patients had severe side effects (three had pneumonia and one had persistent leukopenia and anemia) and could not complete the course. Leukopenia developed in three patients, which was responded to GM-CSF (leukomax). Ponticelli *et al.*, reported infections in five patients, leukopenia in four patients, diabetes in two patients, anemia in four, and neoplasia in one patient who had received a cumulative dose of Chl of 0.9 g out of 27 patients. Except this, they concluded that six-month course of therapy was generally well tolerated, and in long term no major toxicity was noted.[7] On the other hand Latta, *et al.*, in their meta-analysis with CYC and Chl, reported leucopoenia in one-third of patients, severe bacterial infections in 1.5% of the patients under CYC, and in 6.8% under Chl. Seizures were observed in 3.6% of children treated with Chl. Malignancies were observed in 14 children after high doses of either drug.[3] In contrast, in our study, no patient developed seizure and malignancy till the last follow-up. To conclude about the occurrence of malignancy it needs a long-term follow up. Mendoza *et al.*, in spite of very aggressive approach found relatively mild side effects.[12] Tune *et al.*, who also approached aggressively, found slowed growth in four, small cataracts that did not interfere with vision in five, and infections in two (cellulitis and herpes zoster) patients. There were no cases of abdominal striae, DM, or aseptic necrosis of bone.[13] Side effects in different studies are presented in Table 9.

**Table 9:** Side effects in different studies

Side effects	Ponticelli (*N* = 27)	Tune (*N* = 32)	Lata *et al*. (*N* = 1504)	NIMS (*N* = 28)
Infection (%)	18.51	6.25	6.8	21.42
Pneumonia (*n*)	3	Nil	-	3
Herpes zoster (*n*)	1	1	-	2
Cellulitis (*n*)	1	1	-	Nil
Growth retardation (*n*)	Not mentioned	4	Not mentioned	1
Leukopenia (*n*)	4	Not mentioned	1/3rd of patients	3
Anemia (*n*)	4	Not mentioned	Not mentioned	1
Diabetes (*n*)	2	Nil	Not mentioned	1
Malignancy (*n*)	1	Not mentioned	14 children	Nil

Thus, in our study, the overall response rate using Ponticelli protocol in SRNS with FSGS was comparable to that reported previously. This therapy can be effective partially in SR/FR NS with MCD, MPGN, and MesPGN, and ours is probably the first study with these GN. Long-term controlled studies are needed to confirm this. Though side effects are minimal, caution should be taken for leucopoenia and infections such as pneumonia. Long-term follow up is needed to know about malignancy as a side effect. Hence, we conclude that Ponticelli regimen is effective and safe in achieving remission in patients with various types of GN, who were resistant to the usual conventional immunosuppressive drugs. To confirm this, a controlled, multicenter trial of this protocol may be needed.

### Definitions

Complete remission (CR): A decrease of urinary protein excretion to at least 200 mg/day or < 4 mg/m^2^/h in children for at least one month duration with plasma creatinine stable at < 1.5 mg/dl.

Partial remission (PR): A reduction in the rate of urinary protein excretion to between 0.21 and 2 g/day for at least one month duration with plasma creatinine stable at < 1.5 mg/dl.

Relapse: Reappearance of proteinuria to >0.2 g/day in a patient who had either CR or PR.[6]

Renal failure: Persistent doubling of plasma creatinine over the baseline values.[5]

No change: Was considered to have taken place if protein excretion was >2 g/day and the level of plasma creatinine was stable or increased by < 50% over basal value.[7]

Time of response: The number of days from the start of treatment to the first day of CR or PR.[8]

Duration of disease: Onset of disease to the date of renal biopsy.

Steroid resistant (SR): is present in children when NS persists after 4–6 weeks of therapy with a dose of 60 mg/m^2^ /day of prednisolone or after four months of treatment with prednisolone (1 mg/kg b.w./day) in adults.[9]

Frequent relapses (FR): Initially steroid responsive but relapse at a rate of 2 per 6 months or 3 per 12 months or 6 per 18 months.

Steroid dependent (SD): Initially steroid responsive but relapse during tapering of corticosteroid or within two weeks of discontinuing corticosteroid.

Treatment protocol: MP (1 gm or 30 mg/kg b.w. in children) was given i.v. for three consecutive days, followed by prednisolone (0.5 mg/kg/day) orally for 27 days (Cycle A). Cycle A was followed by Chl (0.2 mg/kg/day) orally for one month (Cycle B). Cycles A and B were continued at alternate months for three times each. Total duration of treatment was six months.[10]
